# An integrated analysis of spinal cord transcriptome and gut microbiome unravel age-associated host-microbiome interactions following spinal cord injury

**DOI:** 10.3389/fimmu.2026.1602745

**Published:** 2026-02-25

**Authors:** Yingli Jing, Fan Bai, Zihan Li, Qiuying Wang, Yan Li, Weijin Liu, Shuangyue Zhang, Chen Gao, Yan Yu

**Affiliations:** 1 China Rehabilitation Science Institute, China Rehabilitation Research Center, Beijing Key Laboratory of Neural Injury and Rehabilitation, and School of Rehabilitation Medicine, Capital Medical University, Beijing, China; 2 Center of Neural Injury and Repair, Beijing Institute for Brain Disorders, Beijing, China

**Keywords:** gut microbiome, immune response, neurological recovery, spinal cord injury, spinal cord transcriptome

## Abstract

**Background:**

Spinal cord injury (SCI) leads to irreversible neurological deficits, with emerging evidence highlighting the pivotal regulatory role of gut microbiota in neural repair through the bidirectional gut-brain axis. This study investigates age-related differences in SCI progression by longitudinally profiling multi-omics signatures in young versus aged mice, integrating spinal cord transcriptomics with gut microbiome analysis.

**Methods:**

A traumatic SCI model was established at the thoracic level 10 in mice. The gut microbiota was analyzed through 16S rRNA sequencing. Spinal cord gene expression was profiled using transcriptome sequencing. Correlation analysis was performed to evaluate associated between gut microbiota shifts and differential cytokines expression.

**Results:**

Aging significantly altered spinal cord gene expression profiles after SCI, KEGG pathway analysis revealed that differentially expressed genes (DEGs) in young and aged SCI mice were highly similar, predominantly involving immune and inflammatory response pathways. The age-dependent upregulation of inflammatory cytokines were observed under both sham and post-SCI conditions. Additionally, aging was associated with distinct shifts in gut microbiota composition across different phases of SCI. The abundance of certain bacterial genera, such as *Lactobacillus* and *Dubosiella*, which was significantly reduced in the acute phase, continued to decline in an age-dependent manner during the chronic phase. Correlation analysis indicated that alterations in the abundance of the gut microbiota were closely associated with variations in spinal cord inflammatory cytokine levels.

**Conclusion:**

This study delineates host-microbiome interactions in SCI and sheds light on potential mechanisms underlying age-related impairment of neural repair capacity.

## Introduction

Spinal cord injury (SCI) disastrously damages neural tissues, leading to permanent loss of neurological functions that dampen patients’ quality of life (1–3). Although decades of research have been conducted, with numerous clinical trials undertaken, no safe and effective therapies have been developed to repair damaged spinal cords (2, 4). The global incidence of SCI increases with age, particularly in China, where the incidence rate of elderly people over 70 years old rises sharply (5).With aging, the axonal regeneration ability of peripheral and central nervous system damage decreases, yet the cellular and molecular mechanisms remain unclear (6–8). A better understanding of these mechanisms can identify other molecular strategies to counteract the aging dependent decline in regenerative capacity.

SCI triggers primary and cascaded secondary injury, including ischemia, hypoxia, apoptosis, demyelination, inflammatory cell infiltration and cytokine release and so on (9, 10), among which the immune response is a critical element to hinder recovery after SCI (11–13). Over the past decade, research on the “microbiome-gut-brain” axis has provided new insight into the treatment of SCI (14). Our previous study, along with research by kigerl et al., had reported that gut dysbiosis after SCI deteriorated neurological functional repair (15, 16). Compared to the younger state, elderly people exhibit a series of changes similar to pathological conditions (6, 7, 17). A growing number of studies showed that the development of unique mechanisms preceding injury, combined with injury-specific signals, would drive neurological function toward regenerative failure (6, 7, 18). Thus, promoting spinal cord repair requires a comprehensive understanding of how the immune response and gut microbiota dynamically influence SCI progression across different phases of injury.

Here, we leveraged RNA sequencing to uncover the alteration of transcriptomic signatures in the injured spinal cord from young and aged mice during different phases of injury. Additionally, we also utilized 16S rRNA sequencing - a standard method for profiling complex microbial communities based on sequence variations in conserved bacterial genes - to reveal the alteration of gut microbiota across acute and chronic phases after injury in young and aged mice. Correlation analysis between gut microbiota and differential cytokines suggested potential interactions between the two elements following SCI. Our findings provide a comprehensive characterization of immune aging processes and further elucidate the potential involvement of gut microbiota in tissue repair. These insights are valuable for developing therapeutic strategies that enhance reparative mechanisms while minimizing detrimental effects.

## Materials and methods

### Animals and ethics statement

Young (2 month old) and old (22 month old) female C57BL/6N mice were obtained from the Center of Experimental Animals, Capital Medical University (Beijing, China). All mice were housed in an air-conditioned room where the temperature was 22 ± 2 °C and relative humidity was 55% ± 10%, and followed a standard 12:12 light/dark cycle. Food and water were available ad libitum. We euthanized mice by cervical dislocation after they were deeply anesthetized with 5% isoflurane inhalation, confirmed by the absence of a pedal withdrawal reflex upon firm toe pinch. The animal protocols were approved by The Animal Care and Use Committee of Capital Medical University. (Approval No.: AEEI-2022-157).

### SCI

We used 2% isoflurane for mouse anesthesia. Thereafter, laminectomy was performed to expose the T10 spinal cord, and 70-kilodyne contusion was performed using an Infinite Horizons Impactor (Precision Systems & Instrumentation, Lexington, KY, USA) before suturing of muscles and incision. Throughout the surgery and post-anesthesia recovery, the animals were put into the warming chamber until complete consciousness was reached. The mice were hydrated using Ringer’s solution (0.5 ml, SC) for 5 days postoperatively. The urinary bladder was manually emptied twice or more daily throughout our study period. Surgical interventions and postoperative animal care were conducted following guidelines and relevant policies for rodent survival surgery released by the Experimental Animal Committee of the Capital Medical University. The schematic diagrams of the spinal cord injury modeling process and the experimental groups were shown in **Supplementary Figure 1**. Mice were separated into 6 groups with 8 mice in each group: sham (Y-sham and O-sham); acute phase following injury (3 days post injury, Y-SCI-A, and O-SCI-A), chronic phase following injury (28 days post injury, Y-SCI-C and O-SCI-C). Y: young mice (2 months); O: old mice (22 months); A:acute phase following injury; C: chronic phase following injury.

### RNA extraction and sequencing

The total RNA was extracted from tissue samples using the TRIzol^®^ Reagent and then subjected to a quality assessment using the 5300 Bioanalyzer (Agilent). The extracted RNA was quantified using the ND-2000 (NanoDrop Technologies). The high-quality RNA samples (OD260/280 = 1.8-2.2, OD260/230 ≥ 2.0, RIN ≥ 6.5, 28S:18S ≥ 1.0, > 1 µg) were then used for library construction. RNA purification, reverse transcription, library preparation, and sequencing were performed at Shanghai MajorBio Bio-pharm Biotechnology Co. Ltd. by following the Illumina protocols. The RNA-seq library was prepared using 1 µg of the total RNA, and the process comprised mRNA isolation using poly-A selection, cDNA synthesis using random hexamer primers, and end-repair using ‘A’ base addition. Size-selected libraries (300 bp) were then PCR amplified and sequenced on a NovaSeq 6000 sequencer (2 × 150 bp).

Raw paired-end reads were subjected to quality trimming and control using fastp with default parameters. The clean reads were then aligned to the reference genome using HISAT2 in orientation mode. The mapped reads were assembled using String Tie in a reference-based manner. The threshold for selection was |log2FC| ≥ 1 and FDR ≤ 0.05. Differentially expressed genes (DEGs) were identified using the DESeq2 package in R. KEGG enrichment analysis was performed using the SciPy library in Python. The above data analysis was conducted using the free online Majorbio Cloud Platform (http://www.majorbio.com). RNA sequencing data have been deposited in Sequence Read Archive (SRA) with the accession number PRJNA1241889.

### 16S rRNA gene amplicon sequencing

Fecal samples were collected in 2.0 mL sterile tubes, snap frozen in liquid nitrogen, and stored at –80 °C for further analysis. The fecal samples were then placed in dry ice and sent to Shanghai Majorbio Bio-pharm Biotechnology Co., Ltd. (Shanghai, China) for 16S rRNA Gene Amplicon Sequencing and Bioinformatics and Statistical Analysis. This gene contains conserved and variable regions, where the conserved regions facilitate amplification with universal primers, while the sequence differences in the variable regions serve as molecular markers for identifying bacterial species and genera, making it a standard method for studying the structure of complex microbial communities. Briefly, microbial genomic DNA was extracted using the OMEGA Stool DNA Kit (D4015-02, Omega Bio-Tek, Norcross, GA, USA) according to the manufacturer’s instructions. The quality of extracted DNA was examined by agarose gel electrophoresis, and quantified using QuantiFluor™ -ST (Promega, USA). PCR amplification of the bacterial 16S rRNA genes V3–V4 region was performed using the forward primer 338F (5′-ACT CCT ACG GGA GGC AGC A-3′) and the reverse primer 806R (5′-GGA CTA CHV GGG TWT CTA AT- 3′). After the individual quantification step, amplicons were pooled in equal amounts, and paired-end sequenced (2 × 300 bp) on an Illumina MiSeq platform according to the standard protocols. The raw reads were deposited in Sequence Read Archive (SRA) with the accession number PRJNA1242401.

Alpha/beta diversity analyses, RDA, heatmap generation, and statistical tests based on community data were performed in the QIIME2 and R environment. Data analysis was carried out on the free online Majorbio Cloud Platform (http://www.majorbio.com).

### Statistical analysis

The data are presented as the means and standard errors of the means and were analyzed via SPSS 17.0 (SPSS Inc., Chicago, IL, USA). Two-way ANOVA was applied to evaluate the main effects of mice age (young vs. old) and injury phase (acute phase vs. Chronic phase), as well as their interaction. And two-tailed unpaired t tests were used for comparisons between two groups. Spearman correlation analysis was used to assess correlations. All the statistical tests were performed via GraphPad Prism 9.0 software (San Diego, CA). A significance level of p < 0.05 was considered statistically significant.

## Results

### Aging leads to altered gene profiles in spinal cord tissue after SCI

**Figure 1:**
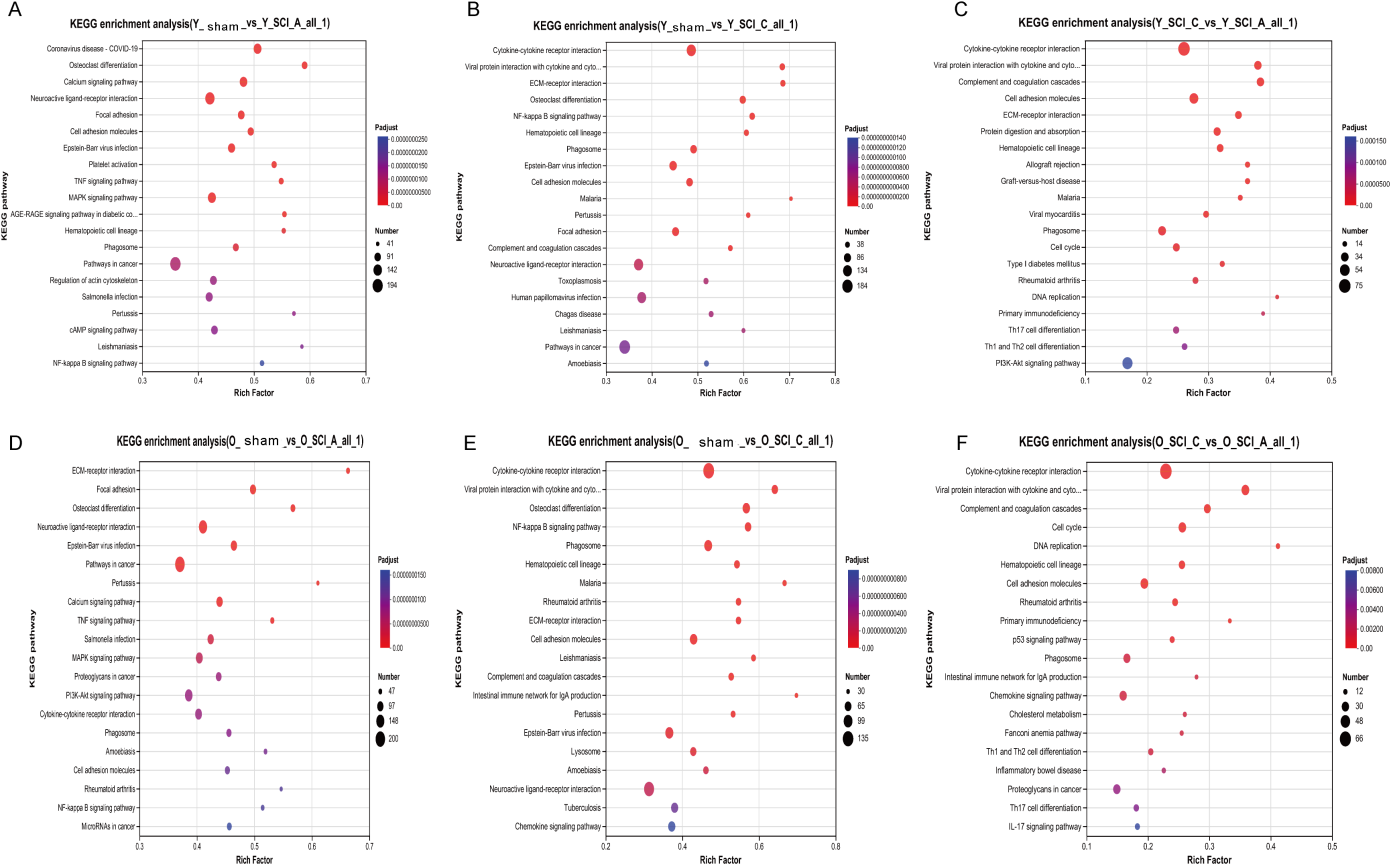
Aging-related alterations in the gene expression of the SCI mice. **(A)** KEGG enrichment analysis between Y-sham and Y-SCI-A. **(B)** KEGG enrichment analysis between Y-sham and Y-SCI-C. **(C)** KEGG enrichment analysis between Y-SCI-A and Y-SCI-C. **(D)** KEGG enrichment analysis between O-sham and O-SCI-A. **(E)** KEGG enrichment analysis between O-sham and O-SCI-C. **(F)** KEGG enrichment analysis between O-SCI-A and O-SCI-C. The size of each bubble is proportional to the number of DEGs assigned to the KEGG terms. Y-sham: n = 5; Y-SCI-A: n = 6; Y-SCI-C: n = 6; O-sham: n = 4; O-SCI-A: n = 5; O-SCI-C: n = 5.

Previous investigations have demonstrated that young mice exhibit superior recovery compared to aged mice after mid-thoracic contusion injury (19, 20). To explore the underlying molecular mechanisms, we employed transcriptome sequencing to analyze the differential gene expression profiles between young and aged mice during both acute and chronic phases of SCI. A series of spinal cord samples in the acute stage (3 days post injury, Y-SCI-A, and O-SCI-A), chronic stage (28 days post injury, Y-SCI-C and O-SCI-C) and sham (Y-sham and O-sham) were collected, sequenced, and subjected to bioinformatics analysis. KEGG pathway analysis revealed that in the young group, the differentially expressed genes (DEGs) between Y-sham and Y-SCI-A were significantly enriched in pathways such as neuroactive ligand-receptor interaction, TNF signaling pathway, and MAPK signaling pathway (**Figure 1A**). The DEGs between Y-sham and Y-SCI-C were primarily enriched in cytokine-cytokine receptor interaction, neuroactive ligand-receptor interaction, and NF-kappa B signaling pathway (**Figure 1B**). Meanwhile, the DEGs between Y-SCI-A and Y-SCI-C showed significant enrichment in pathways including cytokine-cytokine receptor interaction and PI3K-Akt signaling pathway (**Figure 1C**). In the aged group, the DEGs between O-sham and O-SCI-A were significantly enriched in neuroactive ligand-receptor interaction, TNF signaling pathway, MAPK signaling pathway, PI3K-Akt signaling pathway, and cytokine-cytokine receptor interaction (**Figure 1D**). The DEGs between O-sham and O-SCI-C were mainly enriched in cytokine-cytokine receptor interaction, neuroactive ligand-receptor interaction, NF-kappa B signaling pathway, and chemokine signaling pathway (**Figure 1E**). Additionally, the DEGs between O-SCI-A and O-SCI-C were significantly enriched in pathways such as cytokine-cytokine receptor interaction, cell adhesion molecules, and chemokine signaling pathway (**Figure 1F**). Overall, the analysis demonstrated that the enriched pathways of DEGs in both young and old groups were highly similar, with a predominant focus on immune response-related pathways.

### Aging-dependent alterations in the RNA sequencing at the same injury phase of SCI

**Figure 2:**
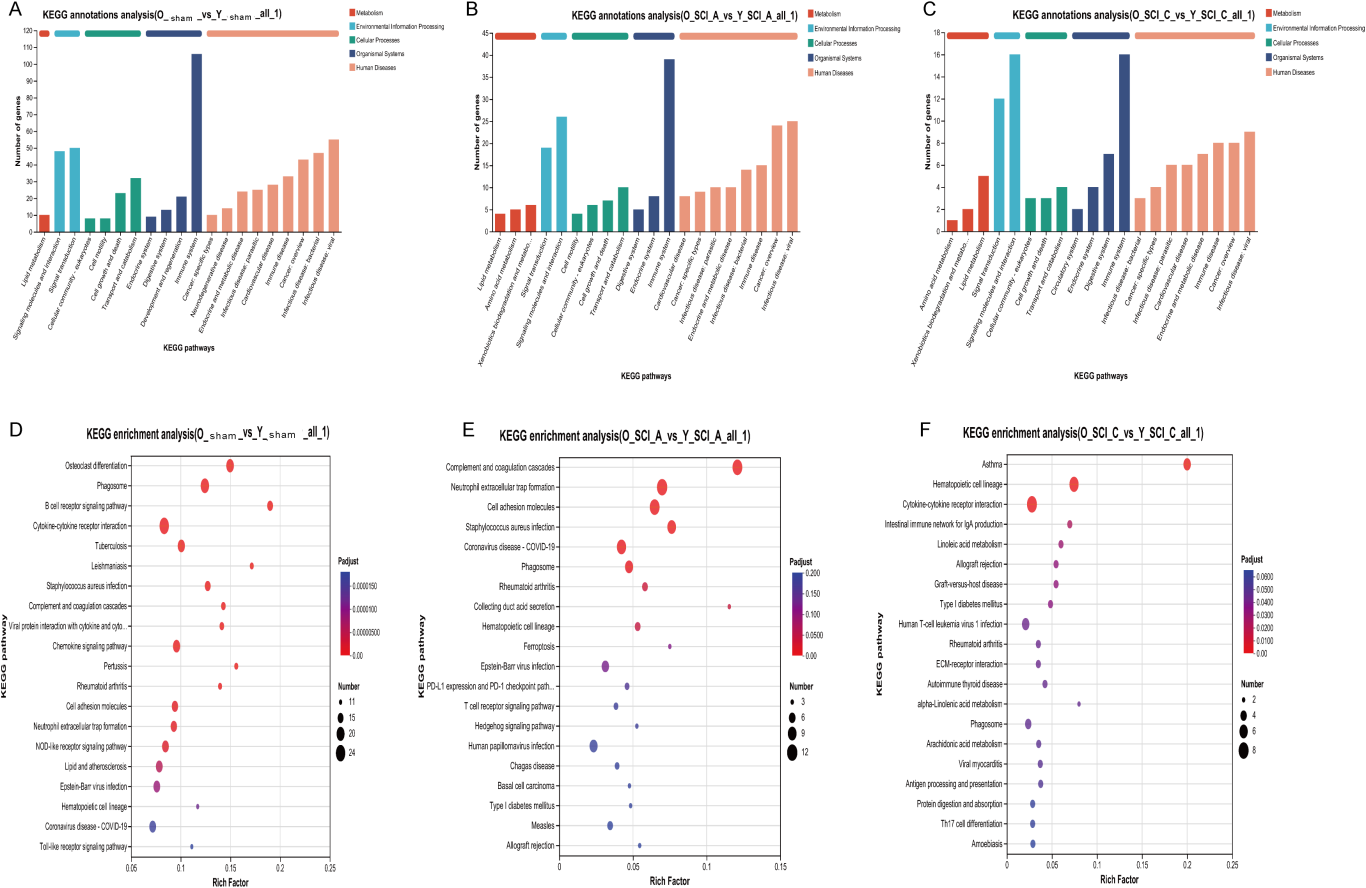
Aging-dependent alterations in the RNA sequencing at the same injury phase of SCI. **(A)** KEGG annotations analysis between O-sham and Y-sham. **(B)** KEGG annotations analysis between O-SCI-A and Y-SCI-A. **(C)** KEGG annotations analysis between O-SCI-C and Y-SCI-C. **(D)** KEGG enrichment analysis between O-sham and Y-sham. **(E)** KEGG enrichment analysis between O-SCI-A and Y-SCI-A. **(F)** KEGG enrichment analysis between O-SCI-C and Y-SCI-C. The size of each bubble is proportional to the number of DEGs assigned to the KEGG terms.

**Figure 3:**
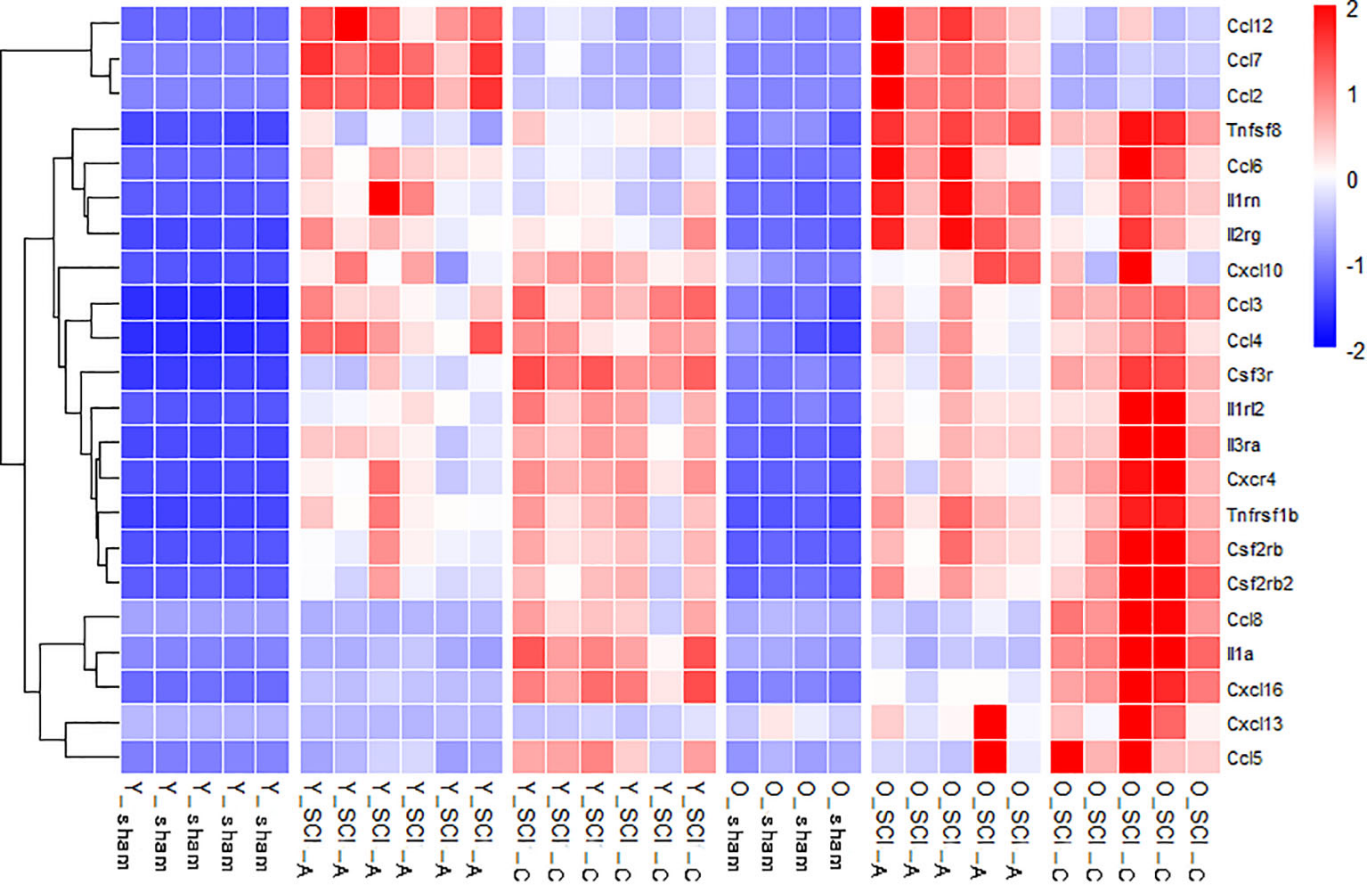
Heatmap of gene expression changes in key cytokines among groups.

To further elucidate the differences in spinal cord gene expression profiles between young and old mice at the same time period after SCI, we performed differential gene expression analysis on the sham, acute phase, and chronic phase groups of young and old mice. KEGG annotation data revealed that, DEGs were primarily enriched in pathways related to the immune system, signaling molecules and interaction, and signal transduction in Y-sham vs O-sham, O-SCI-A vs Y-SCI-A, and O-SCI-C vs Y-SCI-C (**Figures 2A–C**). KEGG pathway enrichment analysis showed that the DEGs between Y-sham and O-sham were significantly enrichment in pathways such as cytokine-cytokine receptor interaction and chemokine signaling pathway (**Figure 2D**). In the acute phase of SCI, the DEGs between O-SCI-A and Y-SCI-A were mainly enriched in pathways including neutrophil extracellular trap formation and cell adhesion molecules (**Figure 2E**). In the chronic phase of SCI, the DEGs between O-SCI-C and Y-SCI-C were significantly enriched in pathways such as cytokine-cytokine receptor interaction and Th17 cell differentiation (**Figure 2F**). Notably, immune responses, particularly DEGs enriched in cytokine-cytokine receptor interaction, showed significant differences in an age-dependent manner following SCI. A heatmap illustrates the expression changes of detected cytokines among groups (**Figure 3**). The heatmap demonstrates that some cytokines were highly expressed in the acute phase (e.g., ccl2, ccl7, ccl12), while others showed relatively higher expression in the chronic phase (e.g., ccl8, ccl4, CXCR4, cxcl13, cxcl16) (**Figure 3**). On the whole, the age-dependent upregulation of cytokines was more pronounced under both normal and pathological conditions.

### Aging-dependent alterations in the gut microbiota at different phases of SCI mice

**Figure 4:**
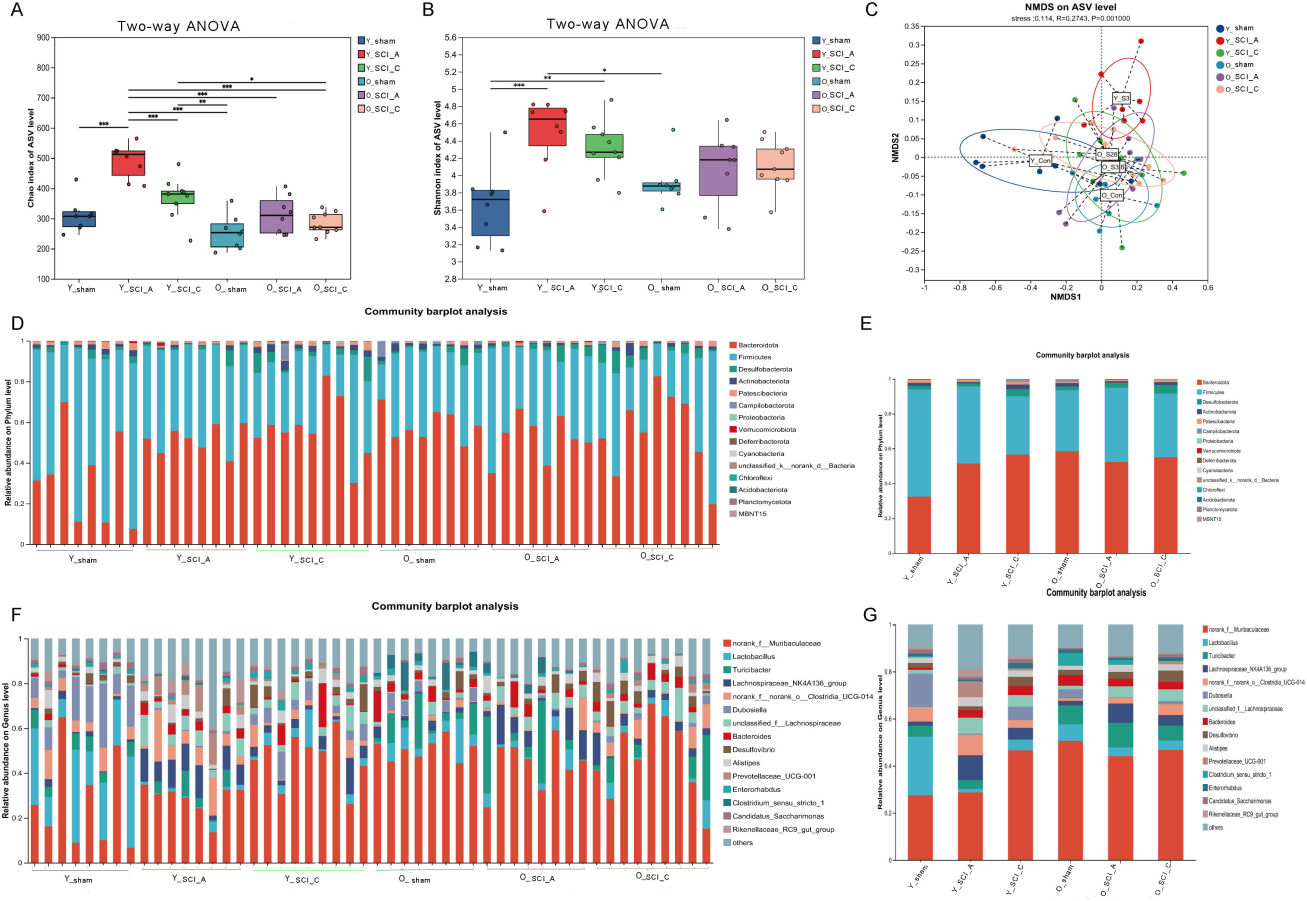
Aging-dependent alterations in the gut microbiota at different phases of SCI mice. **(A–B)** Comparison of the Chao index **(A)** and Shannon index **(B)** based on ASV levels among groups. **(C)** Scatter plots of non-metric multidimensional scaling (NMDS) scores showing the similarity of the bacterial communities on the basis of the weighted UniFrac distance. **(D–E)** Bacterial composition of the different communities at the phylum level. **(F–G)** Bacterial composition of the different communities at the genus level. n = 8/group.

Previous studies showed that the abundance and composition of the gut microbiota are altered, which was observed not only after SCI (16, 21), but also following aging (22, 23). The mechanisms by which injury and aging collaboratively influence gut microbiota changes are yet to be fully elucidated. We explore how gut microbiota is altered in young and old mice after spinal cord injury using 16S rRNA sequencing. As shown in **Figures 4A, B**, significant differences in the Chao and Shannon indices were observed among the groups. The Chao index increased obviously at 3 days after SCI and declined slowly at 28 days after injury in both young and old mice. However, there was a significant difference in Chao index between groups (Y-sham vs Y-SCI-A and Y-SCI-A vs Y-SCI-C) in young mice, whereas there was no statistically significant difference in Chao index between groups of old mice (O-sham vs O-SCI-A and O-SCI-A vs O-SCI-C). A similar trend was observed in the Shannon index. These data suggest that spinal cord injury significantly affects the diversity and the richness of gut microbiota and that the extent of change is more pronounced in young mice after injury compared to old mice. NMDA revealed distinct clustering patterns in the acute phase when compared to the sham group, with the microbiome structures progressively recovering during the chronic phase in young mice. In old mice, the gut microbial structure showed little variation at different time points after SCI compared to the sham group; whereas the clustering pattern of gut microbiota in young mice at 28 days post-injury closely resembled that of old mice (**Figure 4C**). At both the phylum and genus levels, the SCI groups at different stages of injury presented distinct microbial profiles compared with those of the sham group in both young and old mice (**Figures 4D–G**).

**Figure 5:**
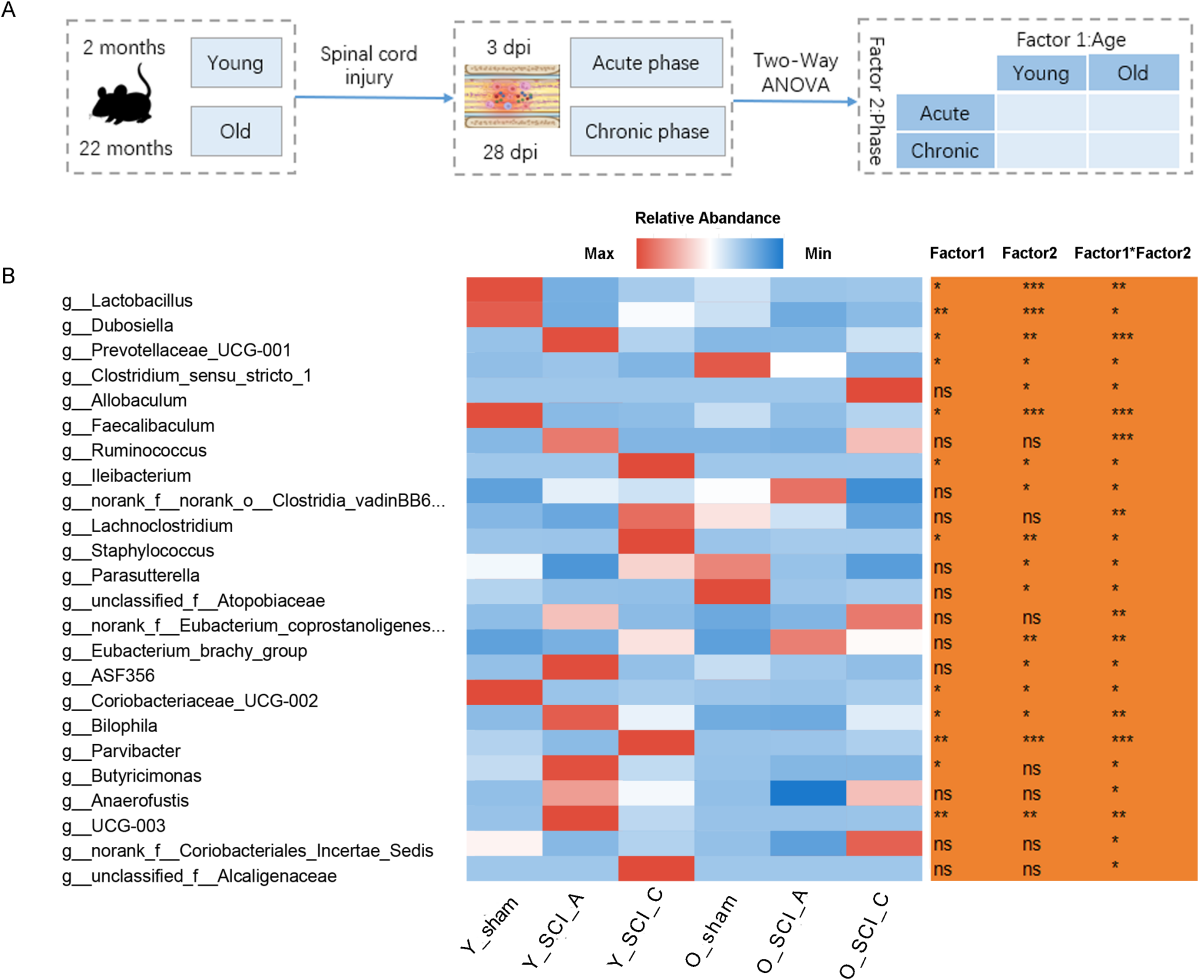
Age induced distinct alterations in microbial abundance following SCI. **(A)** Experimental design. **(B)** A heatmap showing taxa that were significantly regulated by the interaction effect of age (2 months/22 months) and phase (Acute/Chronic) analyzed via two-way ANOVA. *p<0.05; **p<0.01; ***p<0.001.

After SCI, the microbiota in the young and old mice exhibited distinct responses ((**Figure 5**). Centered log-ratio transformation was applied to normalize the microbial data, followed by two-way ANOVA. This analysis identified 24 taxa with significantly different responses to SCI between the young and old (**Figure 5**). The adjusted p values from the two-way ANOVA were shown in **Supplementary Table S1**. The abundance of Lactobacillus, Dubosiella showed an immediate reduction during the acute phase postinjury, followed by a progressive increase throughout the chronic phase; both young and old mice exhibited similar patterns of gut microbiota alterations following SCI. The abundance of g:Prevotellaceae_UCG-001 swiftly increased at 3 days postinjury, followed by a decrease but remained elevated above sham levels until 28 days postinjury, and young and old mice demonstrated comparable changes in their gut microbiota profiles after SCI. Additionally, certain bacterial genera showed distinct variation pattern after SCI in young and old mice. As the condition progresses from the acute to the chronic phase, the microbial composition gradually shifts toward that of the sham group. While, the fluctuation of gut microbiota is significantly greater in young mice when compared to old mice, suggesting that the self-repair capacity of the gut microbiota is diminished in aged individuals.

### Aging-dependent alterations in the gut microbiota at the same injury phase of SCI

**Figure 6:**
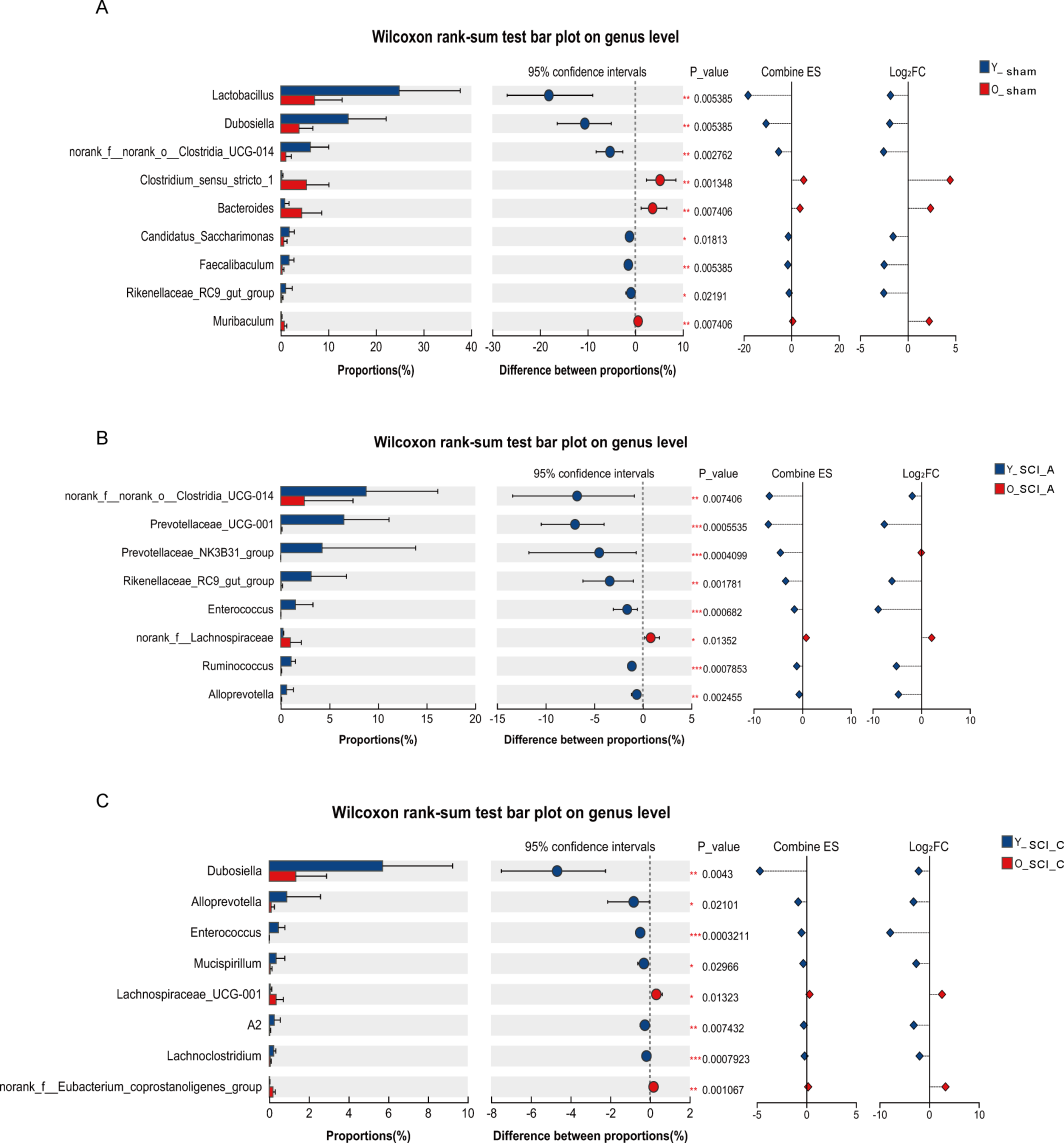
Differential analysis of gut microbial between young and old mice in sham group, acute and chronic phase. **(A)** Quantitative analyses of the relative abundances of bacteria at the genus level between O-sham and Y-sham. **(B)** Quantitative analyses of the relative abundances of bacteria at the genus level between O-SCI-A and Y-SCI-A. **(C)** Quantitative analyses of the relative abundances of bacteria at the genus level between O-SCI-C and Y-SCI-C. *p<0.05; **p<0.01; ***p<0.001.

To further elucidate the gut microbial differences between young and old mice during the same phase after SCI, we conducted differential analysis of gut microbial between young and old mice in sham group, acute and chronic phase. The results showed that compared with Y-sham group, the abundance of *Lactobacillus*, *Dubosiella*, *norank_f:norank_o:_Clostridia_UCG-014*, *Candidatus_Saccharimonas*, *Faecalibaculum* were significantly decreased, while the abundance of *Clostridium_ sensu_stricto_1* and *Bacteroides* were significantly increased in O-sham group (**Figure 6A**). Compared to Y-SCI-A group, the abundance of *norank:f:norank:o:_Clostridia_UCG-014*, *Prevotellaceae_UCG-001*, and *Prevotellaceae_NK3B31*_group was significantly decreased, while the abundance of *norank:f: Lachnospiraceae* abundance was significantly increased in O-SCI-A group (**Figure 6B**). The abundance of *Dubosiella*, *Alloprevotella*, *Enterococcus* were significantly decreased, while the abundance of *Lachnospiraceae_UCG-001* was significantly increased in O-SCI-C group compared to Y-SCI-C group (**Figure 6C**). The results indicate that there are significant differences in the changes of gut microbiota between young and old mice at different injury phases after SCI. Combined with the above analysis of intergroup differences, we noted that *g:Dubosiella*, which was significantly reduced in the acute phase of injury, continued to show an age-dependent decline in the chronic phase as well.

### Correlations between inflammatory cytokines and the microbiota

**Figure 7:**
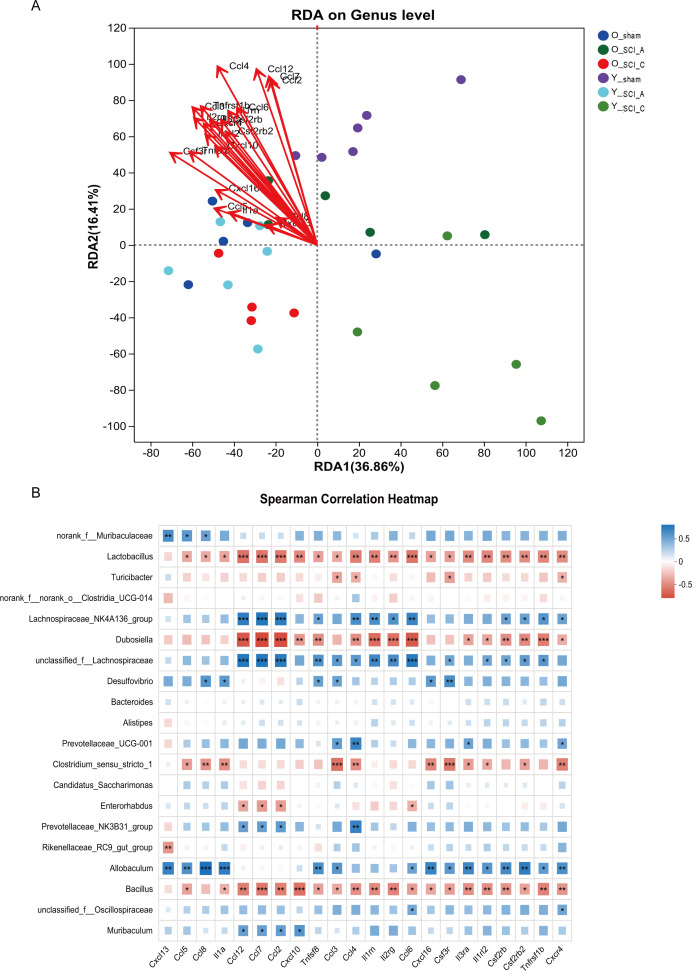
Correlations between differential inflammatory cytokines and the microbiota. **(A)** RDA/CCA revealed that most of the cytokines significantly affected the bacterial composition at the genus level among the groups. **(B)** Correlation heatmap analysis of the effects of the inflammatory cytokines on the community composition of different groups at the genus level. *p<0.05; **p<0.01; ***p<0.001.

To explore the relationship between the host and microbiota, we selected the differential inflammatory cytokines as the environmental factor for RDA. RDA/CCA revealed that most of cytokines significantly affected the bacterial composition at the genus level (**Figure 7A**). Also, the 22 inflammatory cytokines was analyzed by two-way ANOVA, showing 6 inflammatory cytokines (Tnfsf8, Ccl3, Ccl4, Il1rn, Il2rg, Csf3r) with significantly different responses to SCI between the young and old. The adjusted p values from the two-way ANOVA were shown in **Supplementary Table S2**. A correlation heatmap analysis of the relationships between cytokines and the community compositions of the different groups is shown in **Figure 7B**. The abundance of *Lactobacillus*, *Dubosiella*, *Clostridium_sensu_stricto_1* and *Bacillus* showed a significant negative correlation with the majority of cytokines, while the abundance of *Lachnospiraceae_NK4A136_group* and *Allobaculum* exhibited a significant positive correlation with most of the cytokines. Correlation analysis indicated that alterations in the abundance of the gut microbiota were closely associated with changes in the cytokine levels of spinal cord.

## Discussion

In the current study, we use RNA sequencing and 16S rRNA sequencing to explore the pathophysiological mechanisms underlying age-related impairments in spinal cord traumatic injury. Upon contusion, we identified age-related alterations of gene expression profiles and age-related changes in the composition and abundance of gut microbiota. Our study further elucidates a significant correlation between gut microbiota and cytokine networks, providing the evidence for aging-dependent alterations of the microbiota-gut-immune axis following SCI.

Transcriptomic sequencing analyses revealed that differentially expressed genes (DEGs) at distinct injury phases were predominantly enriched in immune response pathways. Furthermore, comparative analyses demonstrated a remarkable similarity in the key signaling pathways associated with DEGs between young and old cohorts, both pre- and post-injury, as well as across different recovery stages following SCI. Subsequent comparative analysis of DEGs between young and old mice within matched injury phases (acute or chronic phase) demonstrated that these DEGs were predominantly associated with immune system processes. Pathway enrichment analysis further revealed significant enrichment of these genes in critical signaling pathways, including cell adhesion molecules and cytokine-cytokine receptor interaction. These findings collectively highlight that age-dependent disparities in SCI are primarily manifested through a more pronounced immune response. A growing number of studies have reported that immune response impedes neurological recovery after SCI by contributing to secondary injury cascade, persistent inflammation and reactive gliosis progression (11–13, 24–27). Shuhui Sun et al. explored the underlying molecular mechanism of primate spinal cord aging by RNA sequencing of bulk samples from young and aged thoracic spinal cord segments. Their data revealed that an downregulation in the expression of genes associated with the production of myelin precursors and neurotransmitter transport and an upregulation in the expression of genes (such as CXCL8, ICAM1, GZMK) linked to pro-inflammatory pathways (28). Andrea Francesca M. Salvador et al. investigated age-dependent immune and lymphatic responses after spinal cord injury by single-cell RNA sequencing (6). They compared naive microglia from the spinal cord in young and aged mice and founded marked upregulation of senescence-associated genes (such as Cxcl16, Ccl2, and C3), with functional enrichment demonstrating predominant involvement in cytokine regulation, chemotactic pathways and so on (6). Luming Zhou et al. reported that CXCL13 was the most prominently up-regulated gene after aged sciatic nerve injury, which was sufficient to recruit CXCR5+B and T cells to the DRG and to impair axonal regeneration (18). In line with the phenotypic observations, our data also showed that the cytokines such as cxcl13, CXCL16, CCL8 were obviously upregulated in aged SCI mice. These data suggested that the cytokines-dominated immune response might serve as novel therapeutic targets for neural repair.

Our research data demonstrated that the gut microbiome undergoes dynamic changes in a time-dependent manner after SCI of both young mice and old mice. Among bacterial genera, the most prominent alterations were the *Lactobacillus* and *Dubosiella*, which showed a similar variation pattern after SCI in young and old mice. The abundance of *Lactobacillus* and *Dubosiella* exhibited a significant reduction following SCI, followed by a gradual recovery over time. However, at 28 days post-injury, the *Lactobacillus* and *Dubosiella* populations still remained substantially lower compared to that in the sham group. In our previous study, a significant reduction in the abundance of Lactobacillus within the intestinal microbiota was observed after SCI (29), and melatonin administration induced a significant elevation in the relative abundances of Lactobacillus which might potentially contribute to the improvements of gastrointestinal function and behavioral outcomes (16). Furthermore, supplementation with *Lactobacillus*-containing probiotics has been shown to enhance *Lactobacillus* colonization and improve the immune microenvironment in favor of locomotory recovery (15). Hao Chang et al. demonstrated that stress-responsive neural pathways modulate gut microbial composition, particularly affecting *Lactobacillus* populations; and the administration of a 12-strain probiotic cocktail (containing Lactobacilli) remarkably decreased the levels of proinflammatory cytokines (e.g., TNF-α, IL-6) (30). These findings collectively suggest that Lactobacillus plays a critical role in maintaining intestinal homeostasis and suppressing systemic inflammation. The depletion might exacerbate neuroinflammation and block locomotor recovery. In APP^swe/^PS1^DE9^ mice, the abundances of Dubosiella newyorkensis were greatly reduced with cognitive decline and age (31). Supplementation with *dubosiella newyorkensis* in aged mice led to a reduction of MDA and the increase of SOD in the serum (32), suggesting the role in oxidative stress regulations. Yanan Zhang et al. found an important role for the *Dubosiella newyorkensis* in rebalancing Treg/Th17 responses and ameliorating mucosal barrier injury by producing short-chain fatty acids, especially propionate and L-Lysine (33).This mechanism complements the immunomodulatory function of Lactobacillus, jointly highlighting the central role of microbial metabolites within the gut-brain axis in maintaining secondary injury and neural repair. Taken together, the observed reduction in beneficial genera like Lactobacillus and Dubosiella likely represents a loss of homeostatic, anti-inflammatory, and barrier-supportive functions, rather than simple interspecies competition. Intervention strategies targeting the beneficial genera — such as drug, specific probiotics, or live bacterial preparations — hold promise for restoring intestinal microecological balance, and modulating systemic immunity. These approaches may offer novel therapeutic avenues for neurological injuries. Future research should further elucidate the specific molecular pathways through which these microbiota and their metabolites influence the central nervous system, and explore their translational potential into clinical interventions.

Evidence has established a robust association between SCI and gut dysbiosis. The focus of attention has shifted to the performance of gut microbiome in immune response after SCI. Kigerl et al. demonstrated that the relative abundance of specific gut microbiota correlates with locomotor recovery in SCI mice, suggesting a critical role for immune cells and cytokines in gut-associated lymphoid tissues (GALTs) during this process (15). Additionally, O’Connor’s study in an SCI rat model revealed elevated expression of inflammatory cytokines (IL-1β, IL-12, MIP-2) associated with alterations in specific gut bacterial taxa (34). In 2021, our team further showed that fecal microbiota transplantation (FMT) intervention in SCI mice effectively reshaped gut microbial composition, suppressed inflammatory responses, and improved both motor function and gastrointestinal outcomes (29). In the present study, differentially enriched high-abundance bacterial genera, especially *Lactobacillus* and *Dubosiella* were significantly negatively related with cytokines altered spinal gene expression. Unlike young animals, aged animals exhibit heightened systemic inflammation even in the absence of injury (6, 8, 17, 35). This chronic inflammatory milieu triggers epigenetic reprogramming of intestinal epithelial cells, thereby perturbing gut microbial homeostasis through dysregulated host-microbiota crosstalk. The resultant dysbiotic microbiota subsequently exacerbates neuroinflammation, ultimately establishing a self-perpetuating inflammation-microbiome interaction loop (36). Emerging evidence indicates that the gut-brain axis dynamically modulates physiological progression following SCI through bidirectional communication mechanisms (15, 29, 37, 38). Importantly, the microbiota-immune-neural triad orchestrated by this axis may unveil novel therapeutic avenues for targeted neurorestorative interventions and aging.

Our results suggest that loss of homeostasis functions of immune microenvironment and microbiome might contribute to the age-related delay in post injury spinal cord repair and long-term functional recovery. To gain deeper insights into the mechanisms underlying age-related functional decline, it is crucial to simultaneously characterize the molecular and cellular signatures of these changes in the spinal cord along with their spatial distribution patterns. The application of spatially resolved single-cell sequencing can provide multidimensional data for such investigations. When elucidating the regulatory roles of specific microbial strains in host disease progression, metagenomic sequencing combined with targeted bacterial culture techniques demonstrates unique advantages. Importantly, integrating multi-state human studies (encompassing normal aging, brain injury, spinal cord injury, and neurodegenerative diseases) with multi-omics analyses in animal models will facilitate systematic elucidation of the molecular mechanisms through which host-microbial interactions contribute to aging and neural repair processes.

## Limitations of the study

This study presents correlative observations linking age-related changes in spinal cord immune gene expression to shifts in gut microbial composition following SCI. While our data suggest an interaction between the gut microbiome and the host immune response within the spinal cord, it is important to note that correlation does not imply causation. The identified associations do not definitively establish a directional or mechanistic pathway from gut dysbiosis to altered intraspinal inflammatory cytokines or vice versa. Furthermore, this study primarily focused on microbial taxonomic composition and host transcriptomic signatures. We did not directly assess functional aspects of the gut barrier (e.g., intestinal permeability, mucosal immunity) or measure microbial-derived metabolites (e.g., short-chain fatty acids), which are crucial intermediates in microbiota-host communication. The absence of these functional data limits our ability to fully delineate the operational mechanisms within the proposed microbiota-gut-immune axis. Future studies incorporating gut functional assays, metabolite profiling, and targeted microbial interventions are warranted to validate the causal relationships and functional implications suggested by the present correlative findings.

## Data Availability

The datasets presented in this study can be found in online repositories. The names of the repository/repositories and accession number(s) can be found in the article/**Supplementary Material**.

## Supplementary material

Supplementary Figure 1 Spinal Cord Injury Modeling Process and Experimental Animal Grouping Schematic. (A) Perform laminectomy. (B) Expose the spinal cord at T10. (C) Spinal Cord Contusion Using IH-400 Impactor. (D) Specific Parameters of the Impactor. (E) Spinal Cord Tissue Post-Injury. (F) Schematic Diagram of Experimental Animal Grouping.

Supplementary Table 1 The adjusted p-values from two-way ANOVA with discriminant taxa.

Supplementary Table 2 The adjusted p-values from two-way ANOVA with differential inflammatory cytokines.
