# Supplementary figures and images for: An integrated analysis of spinal cord transcriptome and gut microbiome unravel age-associated host-microbiome interactions following spinal cord injury
Source: Front Immunol. 2026 Feb 25;17:1602745. doi: 10.3389/fimmu.2026.1602745 (PMC12975597; doi:10.3389/fimmu.2026.1602745)

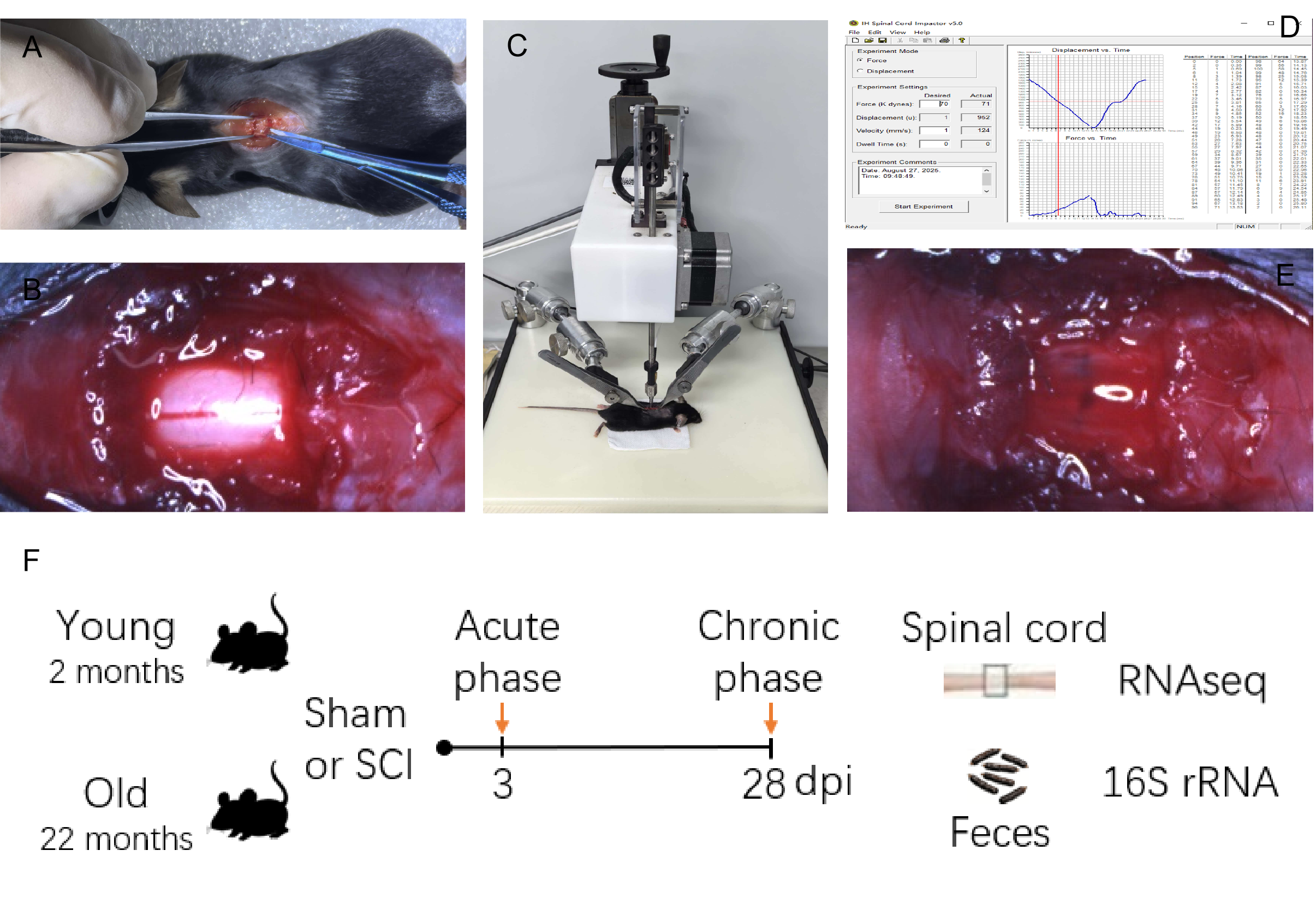

Supplement: Supplementary Figure 1 — Spinal Cord Injury Modeling Process and Experimental Animal Grouping Schematic. (A) Perform laminectomy. (B) Expose the spinal cord at T10. (C) Spinal Cord Contusion Using IH-400 Impactor. (D) Specific Parameters of the Impactor. (E) Spinal Cord Tissue Post-Injury. (F) Schematic Diagram of Experimental Animal Grouping. [file Image1.png]
